# Retraction: ^13^C-CO_2_ pulse labelling evaluation of water deficit on leaf carbon dynamics and whole plant allocation in fruiting coffee

**DOI:** 10.3389/fpls.2026.1985012

**Published:** 2026-09-07

**Authors:** Frontiers Editorial Office

**Please find the retraction statement below:**

The journal retracts the article cited above.

Following publication, the authors contacted the Editorial Office to request the significant corrections of the cited article. An investigation was conducted in accordance with Frontiers’ policies. Given the extent of the corrections, the editors no longer have confidence in the results and conclusions reported. The Chief Editor confirmed that retraction was the appropriate course of action.

This retraction was approved by the Chief Editors of Frontiers in Plant Science and the Chief Executive Editor of Frontiers. The authors agree to this retraction.

